# Doppler Impedance Changes at the Fetal Brain Vessels in a Pregnancy Affected with a Multiple Combination of Uteroplacental Anomalies

**DOI:** 10.1155/2012/293156

**Published:** 2012-02-28

**Authors:** José Morales-Roselló, Núria Peralta Llorens

**Affiliations:** Clínica Morales, C/Trafalgar 46 10 2a, 46003 Valencia, Spain

## Abstract

A fetus with a very rare five-fold combination of uteroplacental anomalies, bicornuate uterus, short cervix with cervical incompetence, multilobed placenta succenturiata, accessory cotyledon within the cervical funneling, and umbilical cord insertion into the anomalous cervical cotyledon, presented an early and marked decrease at the vertebral and middle cerebral arteries Doppler resistances. This cerebral low-impedance state, usually found before labor, and considered an adaptive mechanism developed to protect the fetus at term from labor asphyxia, was present for an unknown reason at 20 weeks. After the patient was treated with vaginal progesterone, the cervix shortening improved and markedly, at the same time, the cerebral vascular resistances increased and maintained an adequate for gestational age impedance until delivery at 34 weeks. As the described uteroplacental anomalies determined a high risk of preterm delivery, due to cervical dilation, cord compresion, and placental haemorrhage, these fluctuating brain vascular changes might be the result of the fetal adaptation to the changes preceding an imminent delivery.

## 1. Introduction

Fetal adaptation to anomalous pregnancy includes not only early lung maturation in growth-restricted fetuses [1] but also a reduction of the cerebral Doppler impedance during chronic hypoxia [2] or preterm labor [3–7]. In the former situation, a fetus with diminished growth and chronic stress advances lung maturation in order to breathe earlier than expected. In the latter, a fetus at risk of severe undernourishment or preterm delivery decreases cerebral vascular resistances, in order to prepare the brain for labor hypoxia. The link between hypoxia and lung maturation has been identified as the action of stress corticosteroids translated into the production of pulmonary surfactant. However, little is known about the relationship between fetal malnutrition, preterm uterine contractions, and decrease of cerebral Doppler impedance. This case describes an example of this last mechanism in a fetus with a high risk of preterm delivery.

## 2. Case Presentation

**Figure 1:**
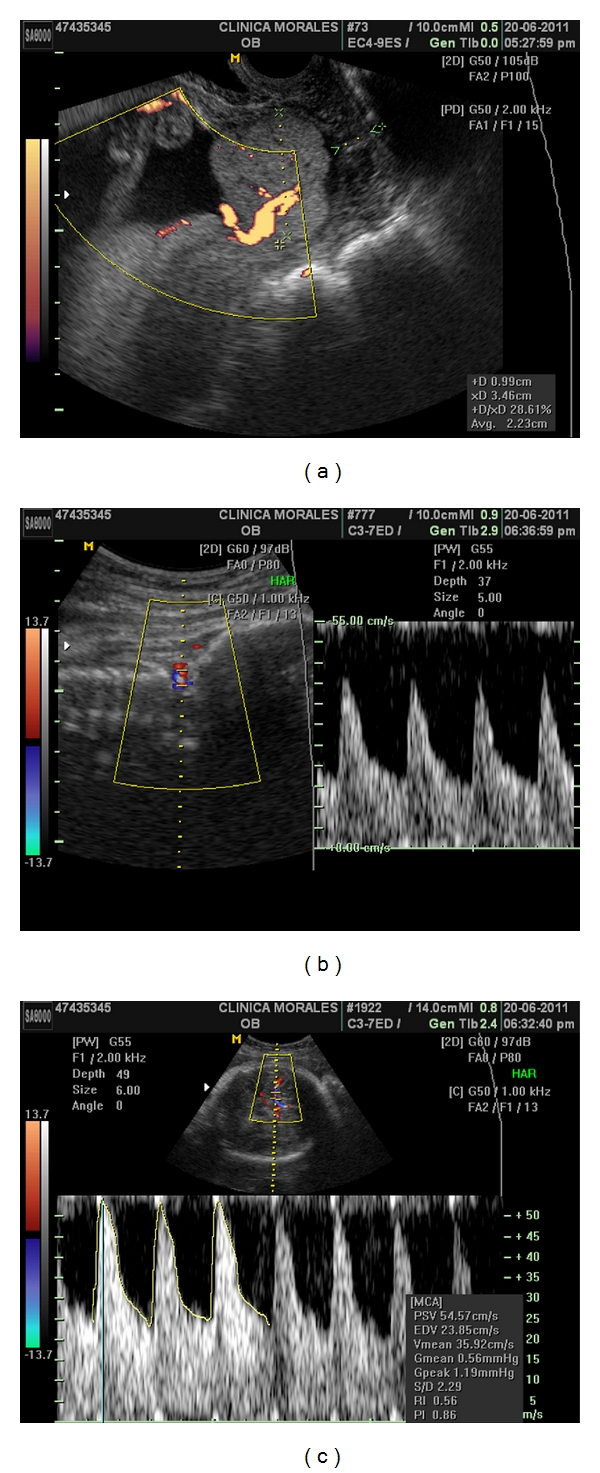
Week 20. Transvaginal colour Doppler ultrasound. The aberrant cotyledon is clearly seen covering the internal os and seems to include the cord insertion. The cervix is very short (9.9 mm) (a) and the Dopper impedances of both the VA and MCA are very low (RI 0.55/PI 0,86) (b, c).

**Figure 2:**
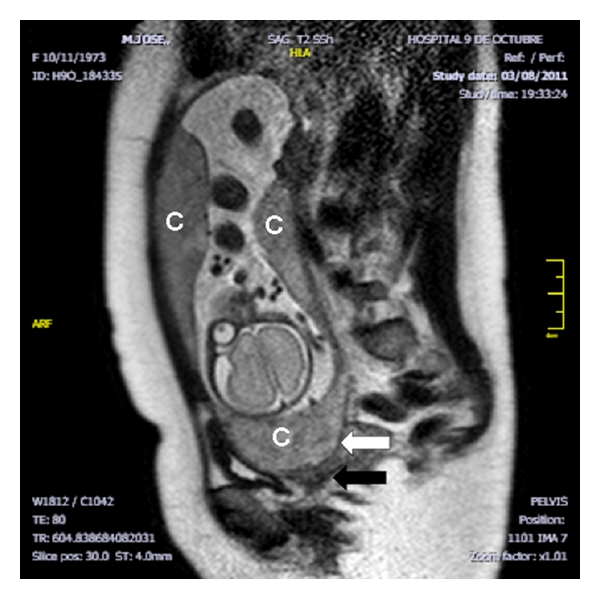
Week 27. Magnetic resonance. One of the three placental portions seen in the image (c) is clearly situated below the fetal head (white arrow) covering the internal os, inside the funnel of a very short and incompetent cervix (black arrow).

**Figure 3:**
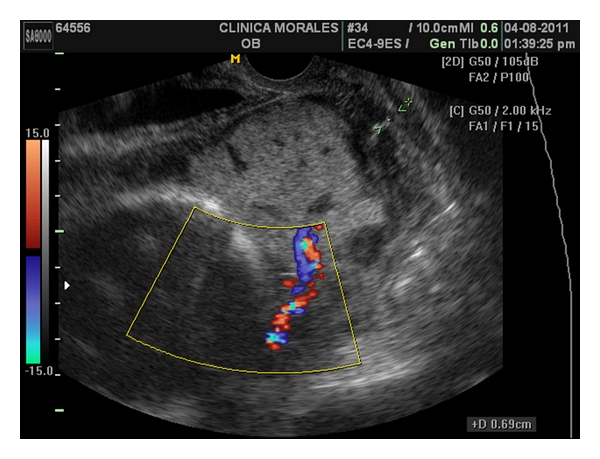
Week 27. Transvaginal colour Doppler ultrasound. The aberrant cotyledon is seen covering the internal os including the umbilical cord insertion. The cervix has become slightly shorter (6,9 mm).

**Figure 4:**
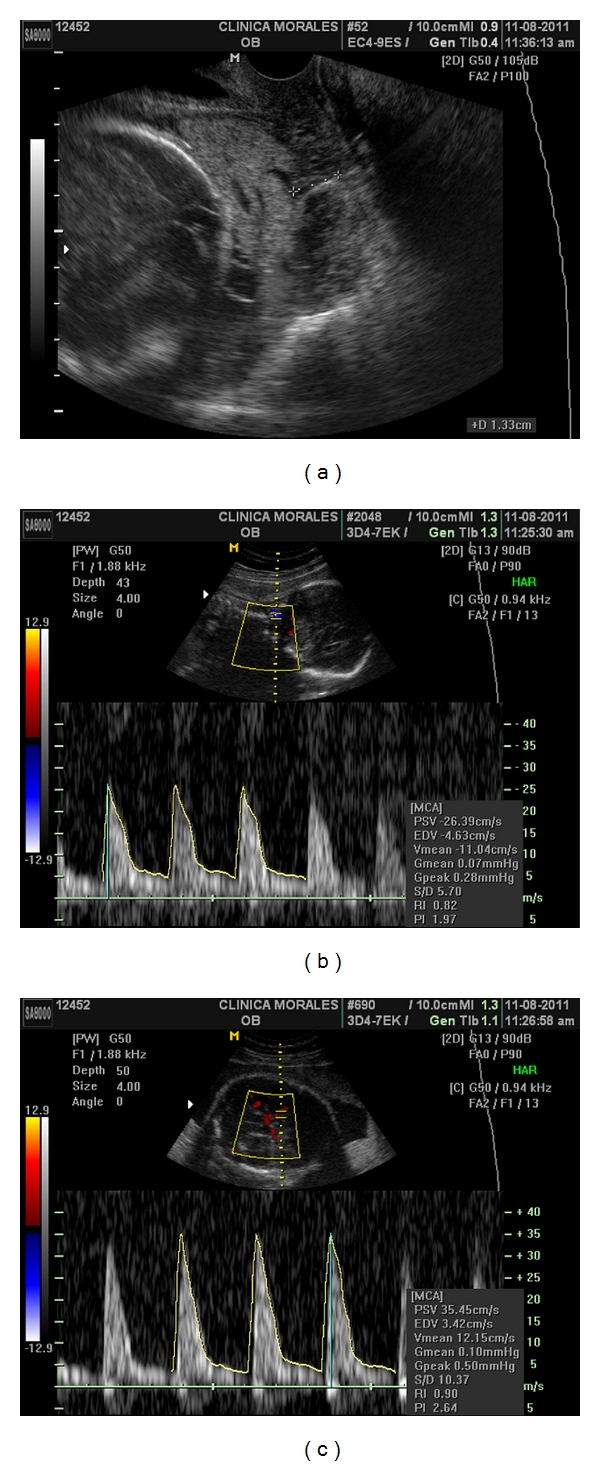
Week 28. Transvaginal colour Doppler ultrasound. The aberrant cotyledon is clearly seen covering the internal os but the cervix has become longer (13 mm) (a). There is a normalization of the VA and MCA impedance (RI 0.82/PI 1.97, RI 0.90/PI 2.64) (b, c).

A 37 years old caucasian woman attended our clinic in her third pregnancy. Her past medical history included: bicornuate uterus, bilateral hypoacusia, a preterm vaginal delivery, and an early miscarriage. The previous follow up was uneventful, with normal blood tests, and low risk Down's syndrome screening. At 20 weeks, the anomaly scan showed a normal fetus with a five-fold set of uteroplacental anomalies: added to the known müllerian anomaly, the ultrasound showed the presence of a placenta succenturiata, with four portions, three main lobes placed in an anterior and posterior position, and one small separated cotyledon identified deep inside the funnel of an incompetent short cervix, which measured only 10 mm. Moreover, the umbilical cord seemed to be inserted into this anomalous cotyledon (Figure 1(a)). Interestingly, despite the fetus maintained an adequate growth and the umbilical artery Doppler was normal, the cerebral vessels showed an early and marked decrease of the vascular impedance in both the vertebral (VA) and the middle cerebral arteries (MCAs) (RI 0.55/PI 0.86, both) Figures 1(b) and 1(c). She was consequently treated with vaginal progesterone 200 mg and was followed exhaustively on a weekly basis in order to achieve the latest gestational age at delivery. At 27 weeks, a magnetic resonance confirmed the sonographic findings (Figure 2) and the colour Doppler ultrasound showed a very shortened cervix (6,9 mm) with an umbilical cord clearly inserted into this cervical anomalous cotyledon (Figure 3). Lung maturation was started with 12 mg of betamethasone in two consecutive days, plus one dose every week, and a cesarean section was planned in case that cervical dilation, placental haemorrhage, or cord compression appeared. Surprisingly, one week afterwards, the cervix became slightly longer (13 mm) (Figure 4(a)). This improvement, perhaps a consequence of the progesterone action, brought along an increase and normalization of the VA and MCA impedance (RI 0.82/PI 1.97 and RI 0.90/PI 2.64) see, Figures 4(b) and 4(c).

**Figure 5:**
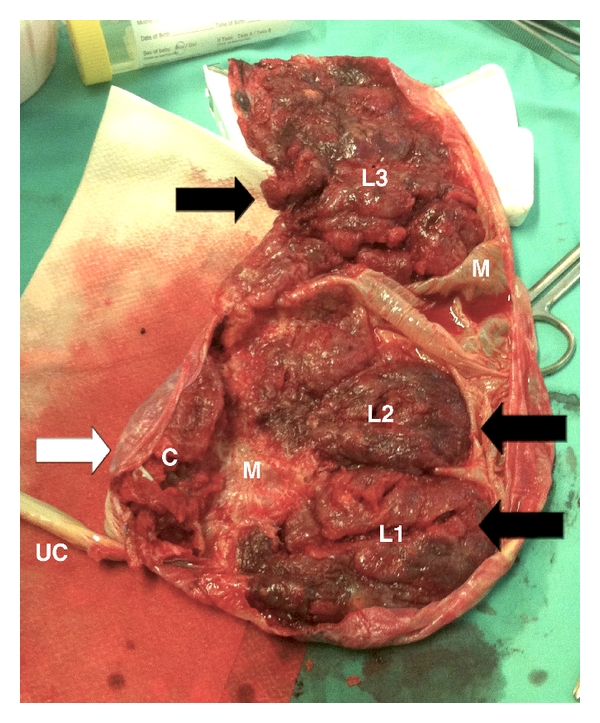
The placenta succenturiata after delivery. An inferior cotyledon may be seen with the insertion of the umbilical cord. Three main lobes, anterior and posteriorly situated, are seen in a superior position. The membranous pars was thick and fibrous. UC: umbilical cord, C plus white arrow: cervical aberrant cotyledon, L1-3 plus black arrows: main lobes, M: membranous pars.

Follow-up with ultrasound and heart rate monitoring showed a fetus with an adequate growth and oxygenation. The cervical length remained unchanged and the high cerebral impedance was maintained until delivery. With an estimated fetal weight of 1900 g, a cesarean section was performed at 34 weeks, delivering a normal 1950 g male newborn, with Apgar 9/10, who was admitted for pediatric control to the neonatal intensive care unit at the 9 de Octubre Hospital and strived properly to date. The placental findings were confirmed after birth (Figure 5). The membranous pars was markedly thick and the lobes and cotyledon had a moderate grade of accretism. Macroscopically, there were three anterior and posterior lobes, and an accessory small cotyledon situated in a much lower position, which included the insertion of the cord.

## 3. Discussion

Placenta succenturiata is a type of multilobed placenta in which one or various cotyledons are separated from the main lobe by membranous areas. This type of placenta is thought to arise after implantation in areas of low perfusion, where the undernourished zones do not develop and become membranous tissue. Membranous vessels may connect the different cotyledons, and the umbilical cord normally inserts into the main lobe, rarely into the accessory cotyledons which use to be atrophic due to ischemic infarction. Depending on the position of the cotyledons and their vascular connections, an association may exist with vasa previa, placenta previa, and retained placental tissue.

Our patient presented this placental anomaly and also a bicornuate uterus, a müllerian malformation which increases the possibility of anomalous placentation and cervical incompetence. However, what made this case interesting was the combination of these two anomalies, together with the existence of an umbilical cord insertion into an accessory cotyledon, which was situated deep in the cervical funnel. As a consequence, the patient presented different obstetrical risks: (1) placenta previa due to the aberrant cotyledon position, (2) umbilical cord prolapse due to the abnormal insertion of the cord deep in the cervical funnel, and (3) preterm labor due to the bicornuate uterus, the incompetent cervix, and the associated anomalies which made delivery at term difficult and risky. Therefore, management of this pregnancy consisted in reaching the most advanced gestational age, just before any possible complication appeared (bleeding, cervical dilation, or initiation of labor with cord compression). As no effective treatment was available, apart from lung maturation with corticosteroids and termination of labor with cesarean section, the patient was treated with vaginal progesterone and controlled weekly. Interestingly, this fetus presented marked changes at the cerebral Doppler impedance which seemed to correlate with the risk of preterm labor. When labor was threatening the fetus at 20 weeks, the fetal response was to initiate a decrease of the cerebral Doppler impedance. When this threat was diminished (probably due to the action of progesterone) the cerebral Doppler impedance returned to normal for gestational age values. Although changes in fetal activity modify the cerebral and umbilical resistances, the vascular changes observed at 20 weeks were too intense, generalized and permanent to be the consequence of such phenomenon. A decrease in the cerebral Doppler impedance has been observed in fetuses before labor in order to prepare the brain for labor hypoxia [2–7]. We postulate that the observed changes in this fetus were the consequence of a similar protective mechanism occurring at a very early gestational age.
